# Alternative Splicing of the Aflatoxin-Associated Baeyer–Villiger Monooxygenase from *Aspergillus flavus*: Characterisation of MoxY Isoforms

**DOI:** 10.3390/toxins10120521

**Published:** 2018-12-05

**Authors:** Carmien Tolmie, Martha S. Smit, Diederik J. Opperman

**Affiliations:** Department of Biotechnology, University of the Free State, Bloemfontein 9300, South Africa; tolmiec@ufs.ac.za (C.T.); smitms@ufs.ac.za (M.S.S.)

**Keywords:** MoxY, aflatoxin biosynthesis, Baeyer–Villiger monooxygenase, hydroxyversicolorone, versicolorone, alternative splicing

## Abstract

Aflatoxins are carcinogenic mycotoxins that are produced by the filamentous fungus *Aspergillus flavus*, a contaminant of numerous food crops. Aflatoxins are synthesised via the aflatoxin biosynthesis pathway, with the enzymes involved encoded by the aflatoxin biosynthesis gene cluster. MoxY is a type I Baeyer–Villiger monooxygenase (BVMO), responsible for the conversion of hydroxyversicolorone (HVN) and versicolorone (VN) to versiconal hemiacetal acetate (VHA) and versiconol acetate (VOAc), respectively. Using mRNA data, an intron near the C-terminus was identified that is alternatively spliced, creating two possible MoxY isoforms which exist in vivo, while analysis of the genomic DNA suggests an alternative start codon leading to possible elongation of the N-terminus. These four variants of the *moxY* gene were recombinantly expressed in *Escherichia coli*, and their activity evaluated with respect to their natural substrates HVN and VN, as well as surrogate ketone substrates. Activity of the enzyme is absolutely dependent on the additional 22 amino acid residues at the N-terminus. Two MoxY isoforms with alternative C-termini, MoxYAltN and MoxYAltNC, converted HVN and VN, in addition to a range of ketone substrates. Stability and flavin-binding data suggest that MoxYAltN is, most likely, the dominant isoform. MoxYAltNC is generated by intron splicing, in contrast to intron retention, which is the most prevalent type of alternative splicing in ascomycetes. The alternative C-termini did not alter the substrate acceptance profile, or regio- or enantioselectivity of the enzyme, but did significantly affect the solubility and stability.

## 1. Introduction

Aflatoxin contamination of key food stocks contribute to the worldwide burden of foodborne disease, with developing countries in sub-Saharan Africa, the Western Pacific, and Asia bearing the highest load [1]. Aflatoxins are fungal, difuranocoumarin-derived secondary metabolites that are synthesised by certain members of the aspergilli, with *A. flavus* and *Aspergillus parasiticus* the most prominent aflatoxin producers [2]. These filamentous fungi colonise an ever-growing list of foodstuffs, both pre- and post-harvest; however, the largest economic and health impact is due to aflatoxin contamination of staple foods, such as maize, peanuts, and rice [3,4,5,6].

Aflatoxins are group 1 carcinogens that primarily target the liver, causing retardation of growth, immune suppression, and hepatocellular carcinoma, with hepatitis B co-occurrence greatly increasing the risk for liver cancer [5,7]. Exposure to aflatoxins causes the disease aflatoxicosis, which can manifest in an acute or chronic state. Although rare, acute aflatoxicosis occurs when heavily contaminated foodstuff (>1000 ppb) is ingested, and can lead to hepatic failure and death [8]. Chronic aflatoxicosis is much more prevalent, especially in developing countries, and is due to long-term aflatoxin exposure, typically by ingestion of contaminated staple foods. Although minimum aflatoxin levels are regulated in commercially sold produce, high levels of aflatoxin contamination have been reported in homegrown crops consumed by subsistence farmers [8].

The biosynthesis of aflatoxins by *A. flavus* and *A. parasiticus* has been studied extensively, in parallel with the synthesis of sterigmatocystin by *Aspergillus nidulans* [9,10,11]. Sterigmatocystin is an intermediate in aflatoxin biosynthesis but is produced as a final product in *A. nidulans* by a truncated version of the aflatoxin biosynthesis pathway. Sterigmatocystin and aflatoxin biosynthesis proceeds via the same intermediates and are catalysed by orthologous enzymes. The aflatoxin biosynthesis pathway is encoded by genes clustered in a 70 kb region that is delimited by a sugar utilisation cluster, and encodes structural enzymes, as well as the transcriptional regulators AflR and AflS [10].

**Figure 1:**
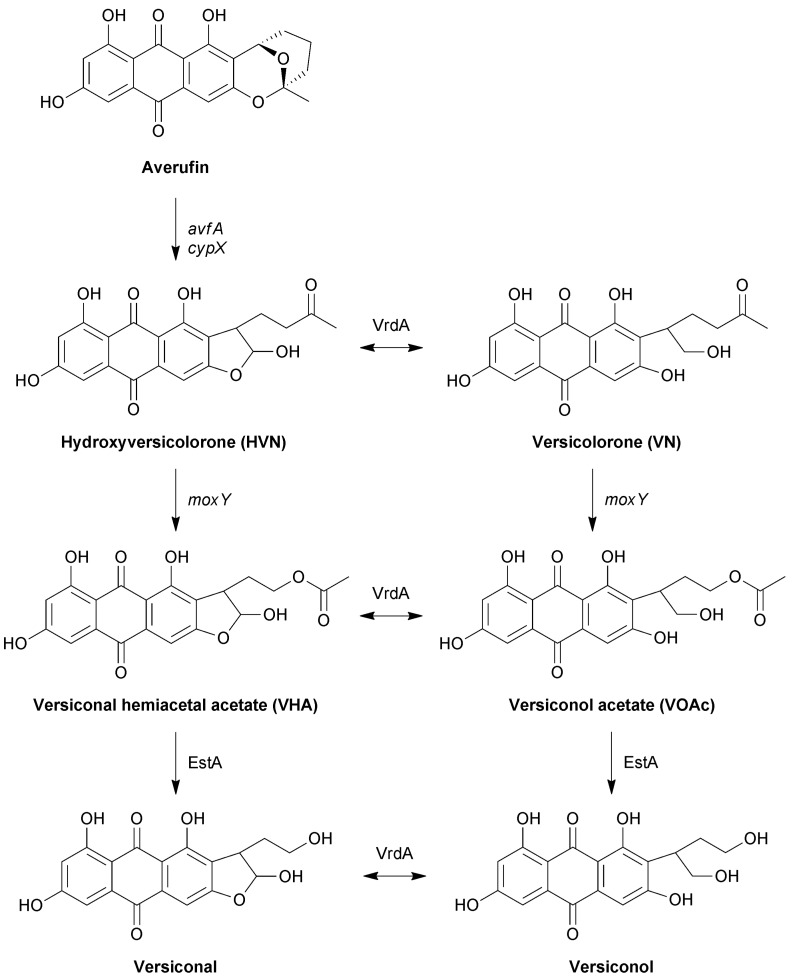
The metabolic grid in the intermediate stages of aflatoxin biosynthesis. Parallel reactions are catalysed by the same enzymes, while the lateral reactions are catalysed by VrdA. Enzymes proven to catalyse reactions by recombinant expression, or by purification from the native host, are indicated in normal case, while genes involved in reactions, as proven by disruption experiments, are indicated in italics.

The intermediate stage of aflatoxin biosynthesis consists of a metabolic grid that operates between averufin and versicolorin B, in which the metabolites exist as oxidised and reduced counterparts at position C 1′ (Figure 1) [12,13]. The lateral reactions in the pathway are mediated by VrdA, an exogenous enzyme that is not encoded for by the aflatoxin biosynthesis cluster. The parallel reactions in the grid are catalysed by the same enzymes, and based on the relative abundance of the metabolites, a main and a side pathway have been proposed. The first reaction in the grid is the conversion of hydroxyversicolorone (HVN) to versiconal hemiacetal acetate (VHA), with the parallel conversion of versicolorone (VN) to versiconol acetate (VOAc) [13]. Metabolite feeding studies showed that HVN is converted to VHA, and VN to VOAc, by an NADPH-dependent cytosolic enzyme [13]. Disruption of the *moxY* gene in the aflatoxin biosynthesis gene cluster led to the accumulation of HVN and VN, with the concomitant loss of aflatoxin production [14]. Feeding experiments showed that these *ΔmoxY* mutants converted VHA and VOAc to aflatoxins, but not HVN or VN. Therefore, MoxY was predicted to serve as a HVN/VN monooxygenase that converts HVN to VHA, as well as VN to VOAc.

The conversion of a ketone to an ester, as in the case of HVN to VHA and VN to VOAc, is a Baeyer–Villiger oxidation that is, in nature, performed by the flavoproteins Baeyer–Villiger monooxygenases (BVMOs). BVMOs use NAD(P)H as electron donor, and molecular oxygen as oxidant, to produce an oxygenated product and water as by-product. BVMOs are widely distributed in nature, where they are involved in the primary metabolism of atypical carbon sources, as well the synthesis of complex secondary metabolites that can be either toxic, or useful, such as antibiotics, anticancer, and antiproliferative agents [15]. However, BVMOs are also intensively studied as biocatalysts that provide a more environmentally friendly alternative to chemical Baeyer–Villiger oxidation, and have superior regio- and enantioselectivity when compared to their chemical counterparts [16].

In this study, we investigate MoxY as the aflatoxin-associated BVMO by recombinant expression of four possible MoxY variants, created by alternative splicing of the gene and elongation of the N-terminus, in *E. coli*. The ability of the MoxY isoforms to convert HVN and VN is evaluated, as well as the conversion of surrogate ketone substrates that are traditionally used to determine the substrate profile, enantio- and regioselectivity of BVMOs.

## 2. Results

### 2.1. Alternative Splicing of the moxY Gene

Initial attempts at heterologous expression of *moxY* in *E. coli* were unsuccessful, as no expression was obtained from the pET-22b(+) vector, whereas expression from the pET-28b(+) vector resulted in inactive enzyme (Figure S1). Despite codon optimisation of the gene for expression in *E. coli* yielding protein from both vectors, soluble expression was still only observed for the pET-28b(+) construct, but the enzyme remained inactive. We postulated that the extended region at the N-terminus, due to the N-terminal His-tag and linker region, improved expression, prompting us to revisit the open reading frame (ORF) annotation of the *moxY* gene. 

**Figure 2:**
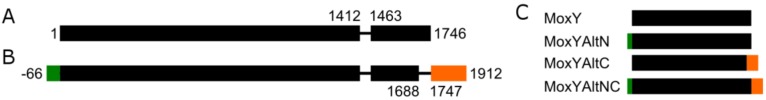
Structure of (**A**) *moxY* and (**B**) *moxYAltNC* genes. The exons are shown as boxes with the introns shown as lines. The common intron to *moxY* and *moxYAltNC* is located at positions 1413–1462, while the second intron in *moxYAltNC* is located at position 1689–1746, splicing out the stop codon and encoding a variant C-terminus, shown in orange. The alternative start codon, 66 bp upstream from the original start codon, leads to the alternative N-terminus, shown in green. (**C**) The four possible variants of the MoxY protein.

Expressed sequence tag (EST) data are sequenced cDNA libraries derived from mRNA and, thus, represent gene expression in vivo. The *moxY* gene, as annotated in the NCBI database, encodes a transcript of 1746 bp (Figure 2A), containing a single intron located at positions 1413–1462, that is spliced out in all the available EST data. However, a region of 58 bp near the C-terminus is spliced as a second intron in three of the seven EST sequences (Figure 2B). This second intron splices out the stop codon, which results in an alternative C-terminus that is both elongated and not conserved with respect to amino acid identity. Additionally, an alternative start codon is located at position −66, resulting in an elongated N-terminus of 22 amino acids. Unfortunately, no EST data is available for the N-terminal region to support the true location of the start codon. An alignment of all the EST sequences to the genomic DNA of the *moxY* gene is shown in Figure S2. The alternative splicing and uncertainty regarding the location of the start codon results in four possible variants of the MoxY protein (Figure 2C): MoxY, as annotated in the NCBI database; MoxY with an elongated N-terminus (MoxYAltN); MoxY with an alternative C-terminus (MoxYAltC); and MoxYAltNC, with both alternative termini.

Sequence analysis revealed that MoxY is a type I BVMO, as it consists of a single polypeptide chain that contains two conserved dinucleotide binding motifs, or Rossmann folds, that belong to the NADP(H)-binding domain and the FAD-binding domain, respectively. Additionally, MoxY has the two characteristic type I BVMO motifs, the “fingerprint” motif at positions 165–177 (FXGXXXHXXXWP) and the second “active site” motif at positions 45–59 (GGXWXXXXYPMXXXD). The FAD-binding domain contains the conserved GXGXXG dinucleotide binding motif, while the corresponding motif in the NADP domain slightly deviates from the consensus (GXGXXS); however, this deviation is also present in cyclopentadecanone monooxygenase (CPDMO) from *Pseudomonas* sp. HI-70 [17], and MO10 and MO18 from *Rhodococcus jostii* [18]. The dinucleotide folds and the signature motifs are critical structural and functional features, and are not affected by the elongation of the N-terminus or the alternative C-terminus of the MoxY variants. 

**Figure 3:**
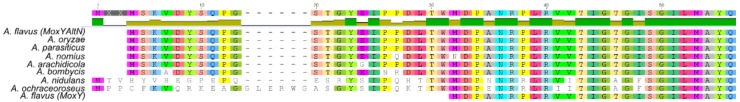
Alignment of the orthologues of MoxY from the aflatoxin- and sterigmatocystin-producing members of the aspergilli, in comparison to MoxYAltN and MoxY from *A. flavus*. NCBI protein accession numbers: *A. oryzae* (XP_001821532.3), *A. parasiticus* (Q6UEF3.1), *A. nomius* (XP_015410220.1), *A. arachidicola* (PIG79656.1), *A. bombycis* (XP_022391198.1), *A. nidulans* (StcW, Q00730.2), and *A. ochraceoroseus* (PTU23613.1).

Multiple sequence alignments of MoxY orthologues, in other members of the aspergilli that produce aflatoxins or sterigmatocystin, were used to determine if these proteins also existed with the elongated N-termini. Orthologues from *A. parasiticus* [19], *Aspergillus arachidicola* [20], *Aspergillus bombycis* [21], *Aspergillus nomius* [22], *Aspergillus ochraceoroseus* [23], the non-aflatoxigenic *Aspergillus oryzae* [24], and StcW from the sterigmatocystin biosynthesis gene cluster in *A. nidulans* [11], as annotated in the NCBI database, all showed elongated N-termini (Figure 3). The termini from *A. oryzae*, *A. bombycis*, and *A. arachidicola* were highly similar to that of MoxYAltN, and contained only 1–3 amino acid substitutions. In addition, the orthologue from *A. nomius* does not contain a second start codon. The termini from *A. nidulans* and *A. ochraceoroseus* are elongated even further than that of MoxYAltN, but are also not conserved when compared to the other orthologues. The aflatoxin biosynthesis gene cluster of *A. ochraceoroseus* is more similar to the sterigmatocystin biosynthesis gene cluster in *A. nidulans* than the biosynthesis gene cluster in *A. flavus* [23], suggesting that “sterigmatocystin cluster-like” MoxY orthologues may vary in sequence from the “aflatoxin cluster-like” MoxY orthologues, but do exist with conserved elongated N-termini.

### 2.2. Cloning and Expression of the MoxY Variants

**Figure 4:**
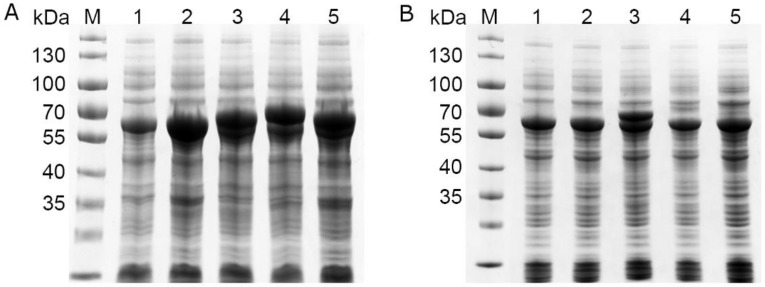
SDS-PAGE analysis of the (**A**) total protein fraction and (**B**) soluble protein fraction of *E. coli* BL21-Gold (DE3) co-expressing the MoxY variants from the pET-28b(+) vector, and the GroES/EL chaperone from the pGro7 vector. M, PageRuler Prestained protein ladder; 1, pET-28b(+) empty vector control; 2, MoxY (66.5 kDa); 3, MoxYAltN (69.0 kDa); 4, MoxYAltNC (73.1 kDa); 5, MoxYAltC (70.7 kDa).

The four possible variants of the *moxY* gene were created by cloning two variants of the gene from genomic DNA and removing their predicted introns through inverse PCR. All four variants of the *moxY* gene expressed in *E. coli* BL21-Gold (DE3) as recombinant enzymes of 66.5–73.1 kDa in size (Figure S3). Partial soluble expression could only be observed for MoxY and MoxYAltN, with no soluble expression observed for MoxYAltNC and MoxYAltC. Co-expression with the molecular chaperone GroES/EL greatly improved the solubility of MoxYAltN, and low levels of soluble MoxYAltNC and MoxYAltC can be observed (Figure 4). Unfortunately, due to overlapping sizes, possible improved solubility of MoxY is obscured by the 60 kDa GroEL component of the chaperone.

### 2.3. Conversion Studies with Hydroxyversicolorone and Versicolorone

**Figure 5:**
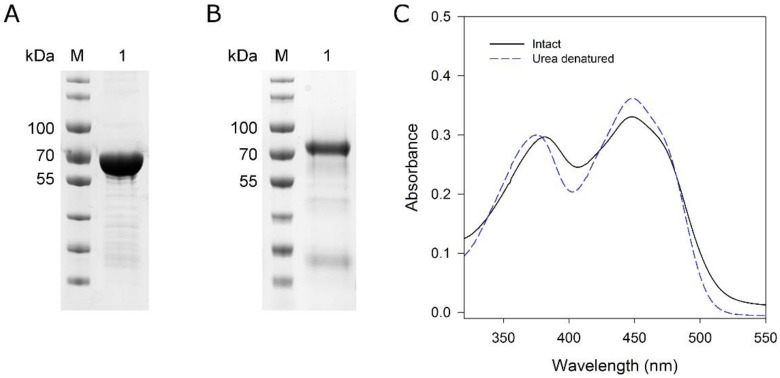
SDS-PAGE analysis of purified (**A**) MoxYAltN and (**B**) MoxYAltNC. M, PageRuler Prestained protein ladder; lane 1, purified MoxYAltN or MoxYAltNC. (**C**) Absorbance spectra of MoxYAltN before and after denaturation with 8 M urea. Extinction coefficient of free FAD at 450 nm: 11.3 mM^−1^ cm^−1^; enzyme-bound FAD at 454 nm: 12.5 mM^−1^ cm^−1^.

To determine which isoform (MoxYAltN and/or MoxYAltNC) converts HVN and VN, biotransformations were performed with the purified enzymes using purified metabolites as substrates. MoxYAltN was purified to near-homogeneity (Figure 5A) using IMAC and size-exclusion chromatography (SEC). Despite extensive incubation with FAD before and after IMAC, only ca. 60% of the MoxYAltN population retained co-factor after SEC. SEC also revealed that MoxYAltN eluted as two peaks, and SEC of a single fraction of the major peak also produced two peaks in a successive round of SEC, while the SDS-PAGE of the peaks showed identical protein profiles (Figure S4). Thus, the purified protein exists as a monomer and, also, either dimerises, aggregates, or unfolds. Absorbance spectra of the purified MoxYAltN sample was used to calculate the concentration of bound FAD in the sample, to ensure that all subsequent biotransformation reactions contained 2 μM of active BVMO, using an extinction coefficient for enzyme-bound FAD of 12.5 mM^−1^ cm^−1^ at 454 nm (Figure 5C). 

MoxYAltNC could be purified to near-homogeneity using IMAC and anion-exchange chromatography (Figure 5B). MoxYAltNC was poorly soluble in the crude extract, and solubility could be slightly improved with the addition of 1% Tween 80 in the lysis buffer. MoxYAltNC remained extensively bound to the GroES/EL complex, thus, a buffer with ATP was used to wash the His-trap column to remove most of the chaperone from the bound MoxYAltNC. Due to the low expression levels and, thus, high volume of cell-free extract loaded on the His-trap column (obtained from 80 g cells), an anion-exchange chromatography step was included to purify MoxYAltNC further to near-homogeneity (Figure 5 and  Figure S5).

MoxYAltNC dissociated completely from the FAD co-factor after IMAC, as well as anion-exchange chromatography, despite extensive incubation with an excess of FAD. Thus, no extinction co-efficient could be determined for MoxYAltNC, and quantification of the active population of the BVMO was not possible. In addition, MoxYAltNC lost ~80% of activity over 12 h (24 h after cell lysis). 

**Figure 6:**
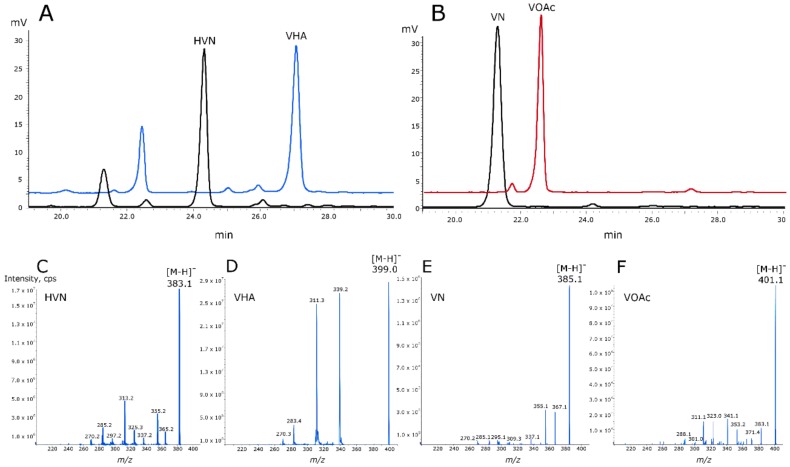
HPLC elution profiles showing the conversion of (**A**) hydroxyversicolorone (HVN) to versiconal hemiacetal acetate (VHA), and (**B**) versicolorone (VN) to versiconol acetate (VOAc). Mass spectra (negative ionisation) of (**C**) HVN, (**D**) VHA, (**E**) VN, and (**F**) VOAc.

Both MoxYAltN and MoxYAltNC fully converted HVN (384 Da) and VN (386 Da) to products with molecular masses of 400 Da and 402 Da, consistent with the insertion of an oxygen atom by BV oxidation, and corresponding to the molecular masses of VHA and VOAc (Figure 6). FAD (50 µM) was included in the biotransformations with MoxYAltNC to enable catalysis.

### 2.4. Substrate Scope of MoxYAltN vs MoxYAltNC

**Figure 7:**
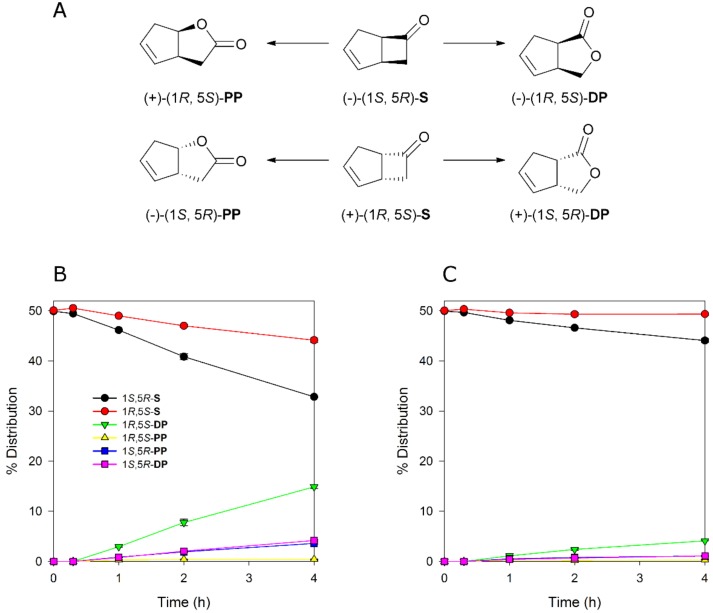
(**A**) Enantio- and regioselective conversion of *rac*-bicyclo[3.2.0]hept-2-en-6-one (S) to the proximal (PP) and distal (DP) products by Baeyer–Villiger monooxygenases (BVMOs). Conversion of *rac*-bicyclo[3.2.0]hept-2-en-6-one by (**B**) MoxYAltN and (**C**) MoxYAltNC over time. Averages of duplicate experiments are shown.

To evaluate if MoxYAltN and MoxYAltNC differ with respect to substrate acceptance, whole-cell biotransformations were performed with surrogate ketone substrates. The bicyclic ketone *rac*-bicyclo[3.2.0]hept-2-en-6-one is considered a “universal” substrate for type I BVMOs, as it is converted by all type I BVMOs except YMOA from *Yarrowia lipolytica* [25]. This substrate, which is a mixture of two enantiomers, is routinely used to characterise the regio- and enantioselectivity of BVMOs. To determine if the alternative C-terminus influences the regio- or enantioselectivity of the enzyme, time-course whole-cell conversions of *rac*-bicyclo[3.2.0]hept-2-en-6-one were performed with MoxYAltN and MoxYAltNC. The two enzymes displayed near identical regio- and enantioselective profiles, although conversions for MoxYAltNC were very low (Figure 7), which may be attributed to the very low level of soluble protein in the cell. The enzymes displayed a preference for the (1*S*, 5*R*) enantiomer, but also converted the (1*R*, 5*S*) enantiomer, and preferentially produced the (1*R*, 5*S*) distal product, with the (1*S*, 5*R*) enantiomers of the distal and proximal products yielded in a ~1:1 ratio, but to a much lesser extent than the (1*R*, 5*S*) distal product. Only trace amounts of the (1*R*, 5*S*) proximal product were detected.

In addition to *rac*-bicyclo[3.2.0]hept-2-en-6-one, 25 other ketone substrates were evaluated for conversion by MoxYAltN and MoxYAltNC (Figure S6 and Table S1). MoxYAltN converted a range of 2-alkanones, with chain lengths ranging from C8–C12, while the 3-alkanone, 3-octanone, was converted to a much lesser extent (Table 1). Regarding the aromatic ketones, MoxYAltN converted phenylacetone and 4-phenyl-2-butanone, as well as 4-(4-hydroxyphenyl)-2-butanone. Of the cyclic and substituted cyclic ketones, MoxYAltN only converted 2-phenylcyclohexanone and, of the bicyclic ketones, converted only *rac*-bicyclo[3.2.0]hept-2-en-6-one. MoxYAltNC displayed an identical substrate profile and regioselectivity in products compared to MoxYAltN, but with much lower conversion rates, which may, once again, be due to the lower expression levels observed. No activity towards any of the substrates was observed for MoxY or MoxYAltC, which may be a consequence of the enzymes being both insoluble and inactive.

### 2.5. Generation of C-Terminally Truncated Variants of MoxYAltN

To investigate why the C-terminus of MoxYAltNC has such an influence on stability and FAD binding, homology models were generated for both MoxYAltN and MoxYAltNC, and FAD binding of the models were evaluated by Ligplot+ (Figure S7). The C-termini of both MoxYAltN and MoxYAltNC were located at the surface of the enzyme, and did not participate in FAD binding. The elongated C-terminus of MoxYAltNC was predicted to adopt some structure in the form of disconnected α helices, but the C-terminal residues were disordered, similar to MoxYAltN. Structural comparisons of MoxYAltN with PAMO [26], CHMO [27], and BVMO_AFL838_ [28] showed that the C-terminus of MoxYAltN is already extended, and an unstructured loop is also present near the C-terminus (Figure S7). In an attempt to improve the stability of MoxYAltN, C-terminally truncated mutants were generated at three positions, residue 501, 545, or 546, and a mutant was constructed from which the loop, spanning residues 528–541, was excised. All the mutants were expressed from the pET-28b(+) vector, but no soluble expression was observed for any of the mutants (Figure S8). When co-expressed with the pGro7 chaperone plasmid, the GroEL component obscured the possible soluble expression of the mutants. However, when their activity was evaluated with whole-cell biotransformations, no conversion of phenylacetone could be observed for any of the mutants.

## 3. Discussion

Secondary metabolism in bacteria and fungi starts with simple starter molecules, which are assembled and transformed into sometimes remarkably complex compounds. The enzymes responsible for the synthesis of these compounds operate sequentially to create a metabolic pathway, and the genes encoding these enzymes are mostly clustered in the genome. In *A. flavus*, aflatoxins are synthesised from acetyl-CoA and malonyl-CoA via the aflatoxin biosynthesis pathway, that is a coordinated effort involving at least 19 enzymatic steps [13,29,30,31]. The genes encoding the enzymes of the aflatoxin biosynthesis pathway are clustered and regulated, resulting in a concerted series of reactions [32]. The pathway requires a great number of oxidation reactions, and the gene cluster encodes a number of oxygenases, including five cytochrome P450 monooxygenases [33] and an anthrone oxidase [34].

We turned our attention to the oxidative conversion of hydroxyversicolorone to versiconal hemiacetal acetate, and the corresponding conversion of versicolorone to versiconol acetate in the side pathway of the metabolic grid in the intermediate stages of aflatoxin biosynthesis (Figure 1). Gene disruption experiments have previously shown that the *moxY* gene, encoded within the aflatoxin gene cluster, is involved in this NADPH-dependent reaction, which is a Baeyer–Villiger oxidation [14]. In nature, BV oxidations are largely catalysed by flavin-dependent Baeyer–Villiger monooxygenases (BVMOs), but can also be catalysed by cytochrome P450 monooxygenases, such as CYP85A2 from *Arabidopsis thaliana* [35], and metal-containing proteins such as toxoflavin lyase in *Paenibacillus polymyxa* strain JH2 [36]. We identified MoxY as a type I BVMO, which consists of a single polypeptide that contains both the NADPH and FAD-binding domains, each with a Rossmann fold motif, as well as the two characteristic motifs for type I BVMOs [37,38].

The *moxY* gene is alternatively spliced, in vivo, and produces two alternative C-termini that differ both in length and sequence. Also, an additional start codon 66 bp upstream from the annotated start codon is present, indicating that the enzyme may exist with an elongated N-terminus, giving rise to four possible variants of the MoxY protein. Although all four variants of MoxY were expressed as recombinant proteins in *E. coli*, the activity of the enzyme is absolutely dependent on the elongated N-terminus, with activity observed only for MoxYAltN and MoxYAltNC.

Multiple sequence alignments with homologues of MoxY from other members of the aspergilli that produce aflatoxins or sterigmatocystin, confirm that the elongated N-terminus of MoxY may be the true active state of the enzyme. Interestingly, the N-terminus does not affect any of the residues that are critically conserved in type I BVMOs. In most of the type I BVMOs, of which the structures have been solved, including CHMO from *Rhodococcus* sp. HI-31 [39], PAMO [26], OTEMO [40], STMO [41], and CHMO from *Thermocrispum municipale* [42], the N-terminus is disordered, and located on the surface of the enzyme. However, the N-terminus of PockeMO [43] is elongated when compared to the structures of the other type I BVMOs, and also adopts structure in the form of disconnected α-helices. Multiple sequence alignments with other known type I BVMOs have shown that numerous BVMOs from both bacteria and fungi have extended N-termini, and, to a much larger extent than MoxYAltN, including HAPMO from *Pseudomonas putida* JD1 [44] and *Pseudomonas fluorescens* ACB [45], CcsB from *Aspergillus clavatus* NRRL1 [46], AusC from *Aspergillus nidulans* FGSC A4 [47], IfnQ from *Streptomyces* sp. RI-77 [48], and BVMO4 from *Dietzia* sp. D5 [49]. Thus, it is possible that this extended terminus plays a crucial structural role in the enzyme.

Both MoxYAltN and MoxYAltNC convert HVN and VN to VHA and VOAc, unequivocally proving MoxYAltN/NC to be the HVN/VN monooxygenases in the aflatoxin biosynthesis pathway of *A. flavus*. The variable C-terminus does not affect the substrate acceptance profile of the enzymes or the regio- and enantioselectivity; however, the alternative C-terminus greatly impacts the solubility, stability, and flavin retention of the protein.

Intron retention is the most common form of alternative splicing in ascomycetes, and may be activated during different physiological states [50,51]. In many cases, intron retention can lead to premature termination by including a stop codon in the ORF, resulting in nonsense-mediated decay. Based on stability and flavin-binding data, MoxYAltN is the dominant isoform of the *moxY* gene. While the mRNA of the more unstable isoform, MoxYAltNC, is generated by intron splicing, the mRNA of MoxYAltN is generated by intron retention.

From the EST data, it can be confirmed that mRNA for MoxYAltN and MoxYAltNC exists during cultivation of *A. flavus*. In general, the mRNA from *A. flavus* undergoes extensive alternative splicing, and can produce multiple isoforms from one gene [52]. EST data, mapped to the whole genome of *A. flavus*, have predicted that 15.4% of gene products are alternatively spliced, although tandem MS experiments contradicted that this is an overestimation [53]. Nevertheless, isoforms are not included in the traditional omics databases, such as RefSeq.

The EST library that was used to predict the alternative splicing of MoxY, was constructed from *A. flavus* grown in eight different media compositions, including one aflatoxin non-inducing medium, and sampled at five different time-points [54]. The EST library is, thus, representative not only of various developmental stages, but also of different regulatory states with respect to nutritional sources. Aflatoxin biosynthesis is a tightly regulated metabolic process, and culture conditions modulate the extent of aflatoxin production [32]. Thus, it is possible that the MoxYAltN and MoxYAltNC transcripts arise in different physiological stages, and do not simultaneously exist in vivo.

Splicing of an intron from pre-mRNA is not only dependent on the sequence within the pre-mRNA, but also the structure of the pre-mRNA, as it can inhibit or promote binding of the spliceosome [55]. The folding of pre-mRNA is also dependent on the rate of transcription, as a slow folding segment in the pre-mRNA may not have sufficient time to fold, in order to be spliced. Thus, it is tempting to speculate that MoxYAltNC may be a result of slow transcription, when conditions for aflatoxin synthesis are not optimal, and will, in turn, result in a short-lived protein as a tool to regulate the flow of metabolites through the aflatoxin biosynthesis pathway. However, the *moxY* orthologue in the aflatoxin biosynthesis gene cluster of *A. parasiticus* does not contain any introns, and the MoxY protein from *A. parasiticus* shares 98.4% similarity (96.3% identity) with MoxYAltN, therefore, *A. parasiticus* does not synthesise MoxYAltNC under any conditions. The true purpose of the alternative splicing of *moxY* thus remains obscure.

In addition to HVN and VN, MoxYAltN/NC convert a wide range of ketones, including aromatic ketones, linear ketones of varying chain length, a substituted cyclic ketone, as well as a bicyclic ketone. The promiscuity in substrate acceptance by MoxYAltN/AltNC is surprising, given the firm physiological role as the HVN/VN monooxygenase in aflatoxin biosynthesis. Due to the highly chromophoric nature of the anthraquinone core of HVN and VN, these substrates could not be used to characterise MoxYAltN/NC spectrophotometrically.

Despite not participating in the core or active site of the enzyme, the C-terminus of MoxY greatly impacts flavin binding and stability. C-terminally truncated mutants, aimed at improving protein stability, were created, as the C-terminus of MoxYAltN is elongated when compared to other BVMOs of which the structures have been solved. However, truncation of the C-terminus resulted in complete loss of function, underscoring the structural role of the C-terminus. BVMOs are highly flexible enzymes that require the movement of domains to position co-factors and shape the substrate binding site [27]. In PAMO, mutation of only two residues distal to the active site induced allosteric-like effects which resulted in an altered substrate profile, indicating a remodelled active site [56]. Thus, the structural effects of the C-terminal portions of MoxYAltN/NC on the active site may be rationalised.

To our knowledge, MoxYAltN/NC are the only type I BVMOs involved in secondary metabolism that also convert a range of surrogate ketone substrates which varies drastically in size when compared to the natural substrate. Other BVMOs have also shown similar malleability in terms of active sites, such as PockeMO [43], which converts small cyclic ketones as well as steroids, and STMO [41], which converts steroids as well as smaller molecules, such as aromatic ketones. Structural studies with BVMOs, co-crystallised with substrates varying largely in size, will provide further insight in how the BVMO active sites can contract and expand to accommodate such a range of diverse substrates.

## 4. Conclusions

The *moxY* gene in the aflatoxin biosynthesis cluster of *A. flavus* encodes a type I BVMO, of which the mRNA is alternatively spliced to create variants with alternative C-termini that have the same substrate profiles and enantio- and regioselectivity, but differ greatly in solubility, stability, and flavin binding. The activity of MoxY is also absolutely dependent on the first 22 amino acid residues of the N terminus, a region that was not annotated on NCBI. MoxYAltN/NC are promiscuous with regard to surrogate ketone substrates, and convert a range of substrates, including linear, aromatic, and substituted cyclic and bicyclic ketones. The role of MoxYAltN/NC in *A. flavus* has been demonstrated by their conversion of hydroxyversicolorone, and versicolorone to versiconal hemiacetal acetate and versiconol acetate, proving MoxYAltN/NC to be the HVN/VN monooxygenases in the aflatoxin biosynthesis pathway.

## 5. Materials and Methods

### 5.1. In Silico Analysis

The *moxY* gene and flanking regions from the aflatoxin biosynthesis cluster of *A. flavus* (accession number NW_002477243.1), as well as the mRNA sequence of the *moxY* gene (accession number gi|238497384), were retrieved from the NCBI database (2009). The genomic sequence of the *moxY* gene was used as query in a BLASTn search against the Expressed Sequence Tag (EST) database, and the resulting nucleotide sequences were mapped to the genomic DNA using Geneious software, version 7.1.3 (Biomatters Limited, Auckland, New Zealand) to identify possible introns. The amino acid sequence of the prototype BVMO cyclohexanone monooxygenase (CHMO) from *Rhodococcus* sp. HI-31 was retrieved from the NCBI database (accession number BAA86293.1, 30 January 2016) [27]. The CHMO sequence was used to identify conserved BVMO residues in the MoxY variants via a local pairwise sequence alignment with EMBOSS-Water [57]. Homologues of the MoxY gene were retrieved from the aflatoxin biosynthesis gene cluster of *A. parasiticus* (nucleotide accession number AY371490.1, amino acid accession number AAS66023.1), the sterigmatocystin biosynthesis gene cluster of *A. nidulans*, (*stcW*, nucleotide accession number U34740.1, amino acid accession number AAC49191.1), and the silent aflatoxin biosynthesis cluster of *A. oryzae* (nucleotide accession number AB196490, amino acid accession number BAE71333.1), as well as homologues from *A. bombycis* (amino acid accession number XP_022391198.1), *A. arachidicola* (amino acid accession number PIG79656.1), *A. ochraceoroseus* (amino acid accession number PTU23613.1), and *A. nomius* (amino acid accession number XP_015410220.1), which were identified by using a blast search with MoxYAltN; and the amino acid sequences were aligned using Clustal Omega [58].

### 5.2. Bacterial Strains and Growth Conditions

*Escherichia coli* TOP10 cells (Thermo Fisher Scientific, Waltham, MA, USA) were used during the generation of the various expression constructs. The pSMART plasmid (Lucigen, Middleton, WI, USA) was used to clone and manipulate the *moxY* genes. *E. coli* cells were routinely cultivated at 37°C in Luria-Bertani medium, and kanamycin (30 g∙L^−1^) was used to ensure maintenance of the plasmid. All restriction and cloning enzymes were purchased from Thermo Fisher Scientific. *E. coli* strain BL21-Gold (DE3) (Agilent Technologies, Santa Clara, CA, USA) was used for protein expression using the pET-28b(+) and pET-22b(+) plasmids (Novagen/Merck, Darmstadt, Germany). The pET-28b(+) constructs were additionally co-expressed with the pGro7 chaperone plasmid (TaKaRa Bio Inc., Kusatsu, Japan). The plasmids were maintained by adding the appropriate antibiotics: kanamycin (30 mg∙mL^−1^, pET-28b(+)), ampicillin (100 mg∙mL^−1^, pET-22b(+)), and chloramphenicol (20 mg∙L^−1^, pGro7).

### 5.3. Fungal Strains and Growth Conditions

*Aspergillus flavus* NRRL3357 was obtained from the Agricultural Research Service culture collection (Peoria, IL, USA). *A. parasiticus* hvn-1, also denoted as WE-47, is a mutant strain accumulating HVN and VN [13,14]. Both strains were maintained on PDA agar at 25 °C and 4 °C.

### 5.4. Cloning of moxY Variants

*A. flavus* NRRL 3357 was cultivated in 100 mL PDB broth for 5 days at 25 °C with shaking (200 rpm). Genomic DNA (gDNA) was extracted using the ZR Fungal/Bacterial DNA MicroPrep kit (Zymo Research, Irvine, CA, USA). Two variants of the *moxY* gene were amplified from the gDNA of *A. flavus* by PCR, namely *moxY* and *moxYAltNC* (Figure 2). The KOD Hot Start polymerase kit (Novagen/Merck) was used to perform all PCR steps, as per manufacturer’s instructions. Primer pairs 1 and 2 were used for the amplification of *moxY* and *moxYAltNC*, respectively (Table 2), and the resulting PCR products were ligated into pSMART. Introns were removed from the pSMART:moxY and pSMART:moxYAltNC constructs by inverse PCR using primers that anneal to either side of the intron, in this manner amplifying the entire plasmid except the intron. The PCR products were digested with *Dpn*I to remove template DNA, followed by phosphorylation and blunt-end ligation to circularise the plasmid. The common intron to *moxY* and *moxYAltNC* was removed using primer pair 3. A pSMART:moxYAltNC construct, from which the first intron was removed successfully, served as template for removal of the second intron by using primer pair 4. The open reading frames were subcloned from pSMART to pET-28b(+) and pET-22b(+) for expression by using *Nde*I and *Xho*I for *moxY*, and *Nde*I and *Hind*III for *moxYAltNC*.

*MoxYAltN* and *moxYAltC* variants were constructed by recombination of the N-terminal portions of pET-28b(+):moxY and pET-28b(+):moxYAltNC plasmids, using *Xba*I and *Eco*RI.

### 5.5. Protein Expression

*E. coli* BL21-Gold(DE3), as well as *E. coli* BL21-Gold(DE3) previously transformed with the pGro7 plasmid, were used to express the pET-28b(+) and pET-22b(+) constructs in ZYP5052 auto-induction media [59] at 20 °C and 200 rpm for 36 h, containing kanamycin (30 mg∙L^−1^) or ampicillin (100 mg∙mL^−1^). Expression of GroES/EL from pGro7 was induced with arabinose (0.5 mg∙L^−1^), and the media was supplemented with chloramphenicol (20 mg∙mL^−1^) to maintain the pGro7 plasmid. Cells were harvested by centrifugation and resuspended in 200 mM Tris (pH 8). Cells were lysed using a One Shot cell disruptor at 30 kpsi (Constant Systems Ltd., Northants, UK). The insoluble fraction was removed by centrifugation at 20,000× *g* and 4 °C for 30 min. Expression levels in both the total and soluble protein fractions were evaluated by SDS-PAGE. PageRuler Prestained Protein Ladder (Thermo Fisher Scientific) was used as molecular weight marker, and proteins were visualised by staining with Coomassie Brilliant Blue R-250.

### 5.6. Whole-Cell Biotransformations with Surrogate Ketone Substrates

Whole-cell biotransformations with ketone substrates were performed using a 1 mL reaction volume in 40 mL amber glass vials. The reaction mixture comprised 200 mM Tris (pH 8), 0.5 g cells, 100 mM glucose, 100 mM glycerol, 10 mM substrate, and 1% (*v*/*v*) methanol. The reactions were initiated by the addition of substrate solubilised in methanol. The range of ketone substrates tested were purchased from Sigma Aldrich (St. Louis, MO, USA) and is depicted in Figure S6. The vials were incubated at 20 °C for 2 h with shaking (200 rpm) after which the reactions were stopped and extracted with 1 mL of ethyl acetate containing an internal standard (2 mM 1-undecanol or 3-octanol). Gas chromatography–mass spectrometry was used to analyse the extracted reaction mixtures on a Finnigan TRACE GC Ultra (Thermo Fisher Scientific) equipped with a FactorFour™ VF-5ms column (60 m × 0.25 mm × 0.25 µm, Agilent Technologies). GC-FID chromatography with an Astec CHIRALDEX™ G-TA column (30 m × 0.25 mm × 0.12 µm, Sigma Aldrich) was used for chiral analysis. Detailed gas chromatography programs are indicated in Tables S2 and S3 (Supplementary Information). Activity was expressed as percentage conversion with averages of duplicate reactions reported.

### 5.7. Purification of MoxYAltN

All purification steps were performed at 4 °C. MoxYAltN was purified using immobilised metal-affinity chromatography (IMAC). Cells were grown and harvested as described, and resuspended in 50 mM Tris buffer, pH 7.4, containing 0.5 M NaCl and 20 mM imidazole (binding buffer). Cells were lysed by single passage through a continuous flow cell disruptor (Constant Systems Ltd.) at 30 kpsi and 4 °C. Crude extracts were obtained by ultracentrifugation at 100,000× *g* for 90 min at 4 °C and the clear supernatant was loaded onto a 5 mL HisTrap FastFlow column (GE Healthcare, Chicago, IL, USA) equilibrated with binding buffer. Unbound proteins were removed by washing the column with ten column volumes of binding buffer. Bound proteins were eluted in the same buffer (100 mL) with an increasing linear gradient of imidazole (0.5 M final concentration). Collected fractions were analysed with SDS-PAGE to evaluate enzyme purity as well as for selective pooling of fractions. The co-factor flavin adenine dinucleotide (FAD) was added in excess to the pooled fractions obtained after IMAC and incubated at 4 °C overnight to ensure maximum co-factor occupancy. MoxYAltN was further purified using size-exclusion chromatography. The pooled fractions were concentrated to 2 mL by ultrafiltration using a 30 kDa NMWL Amicon centrifugal ultrafiltration unit (Merck). Size exclusion of MoxYAltN was performed using a Superdex HR 100 column or a Superdex HR 200 column (GE Healthcare) with 100 mM potassium phosphate buffer, containing 100 mM NaCl.

### 5.8. Purification of MoxYAltNC

Cells were grown and harvested as described, and resuspended in 50 mM Tris buffer, pH 7.4, containing 0.25 M NaCl, 30 mM imidazole, 50 mM KCl, 20 mM MgCl_2_ and 1% Tween 80. Cells were lysed and ultracentrifuged as described for MoxYAltN. The supernatant was loaded onto a 5 mL HisTrap FastFlow column (GE Healthcare) equilibrated with binding buffer (30 mM Tris buffer, pH 7.4, containing 0.25 M NaCl, 20 mM imidazole, 50 mM KCl, 20 mM MgCl_2_). The column was washed with 10 column volumes of binding buffer to remove unbound proteins, followed by 30 column volumes of binding buffer containing 5 mM of ATP to remove the GroES/EL chaperone from the bound MoxYAltNC. Bound proteins were eluted in the same buffer (100 mL) with an increasing linear gradient of imidazole (0.5 M final concentration). Fractions were analysed and pooled as described for MoxYAltN, and incubated with FAD at 4 °C. After IMAC, MoxYAltNC was further purified using anion exchange chromatography. The pooled IMAC fractions were concentrated to 2.5 mL by ultrafiltration using a 30 kDa NMWL Amicon centrifugal ultrafiltration unit (Merck) and buffer exchanged to the anion exchange binding buffer (50 mM Tris pH 8.0 and 50 mM NaCl), using a PD-10 desalting column (GE Healthcare). The desalted MoxYAltNC was loaded onto a Q HP anion exchange column (GE Healthcare) equilibrated with binding buffer. Unbound proteins were washed off with 10 column volumes (50 mL) of binding buffer and MoxYAltNC was eluted with an increasing linear gradient of NaCl. The fractions of interest were once again pooled, concentrated with ultrafiltration and desalted with a PD-10 column to 200 mM potassium phosphate buffer, pH 8.0, containing 100 mM NaCl.

### 5.9. Protein Quantification

Protein concentrations were determined by the Pierce BCA protein assay kit (Thermo Fisher Scientific) using bovine serum albumin as standard. To determine the FAD content, the protein sample of approximately 3.5 mg∙mL^−1^ was incubated with 8 M urea for 40 min, followed by spectrophotometric quantification of the released FAD using the extinction coefficient of 11.3 mM^−1^cm^−1^ at 450 nm, in a DU800 spectrophotometer (Beckman Coulter, Brea, CA, USA).

### 5.10. Purification of Hydroxyversicolorone and Versicolorone

*A. parasiticus* hvn-1 was cultivated in aflatoxin-inducing YES media (2% yeast extract, 20% sucrose) as standing cultures for 7 days at 28 °C. Bright orange mycelial mats were harvested, washed with distilled water, and freeze-dried, after which it was extracted with acetone. HVN and VN were purified from the total metabolite extract by preparative TLC (Silica gel GF UV254, 20 × 20 cm, 1000 µm, Analtech, Sigma Aldrich), resolved twice with a chloroform–ethanol solvent system (95:5). Orange bands with an RF of 0.42 for HVN and 0.47 for VN were extracted. The identities of HVN and VN were verified by comparison to synthetic [1′-2H]HVN and [1′-2H]VN standards, and molecular masses were verified using LC–MS.

### 5.11. Conversion Studies with Hydroxyversicolorone and Versicolorone

The conversion of HVN and VN by MoxYAltN and MoxYAltNC were evaluated by biotransformations with purified enzyme. The reactions were performed in 40 mL glass amber vials with a total volume of 1 mL, for 24 h at 20 °C and 200 rpm. The reactions comprised 130 µM HVN or VN, 5 μM purified enzyme for MoxYAltN and 0.16 mg protein for MoxYAltNC, 0.3 mM NADP^+^, 0.5 U glucose dehydrogenase from *Bacillus megaterium* for co-factor regeneration, 100 mM glucose, in 100 mM potassium phosphate buffer, pH 8. FAD (50 μM) was added to the reactions of MoxYAltNC. The reactions were stopped by adding 1 mL ethyl acetate, the organic phase was dried and resuspended in 300 μL acetonitrile, and analysed with HPLC. Negative controls were performed using the exact reaction makeup, without enzyme. Substrates and products were evaluated with a Prominence HPLC system (Shimadzu, Kyoto, Japan) equipped with a Discovery C18 column (5 μm particle size, L × I.D. 25 cm × 4.6 mm; Supelco, Sigma Aldrich) with an acetonitrile–water solvent system. Retention times of compounds are shown in Figure 6. Molecular masses of substrates and products were identified by LC–MS. Samples were analysed on an ABSCIEX 4000QTRAP hybrid triple quadrupole mass spectrometer with Shimadzu front end. Ten microliters of each sample was injected onto a Discovery HS C18 reverse phase column (250 × 4.6 mm, Supelco) and separated using a 20% to 50% linear 0.1% formic acid (Solvent A) and acetonitrile with 0.1% formic acid (Solvent B) gradient over 30 min at 1 mL/min for a total runtime of 45 min including column re-equilibration. Eluting analytes were analysed in negative APCI ionisation mode using an information dependent acquisition (IDA) method where ions between 380 and 480 Da with intensities above 100,000 counts per second (cps) originating from an enhanced MS (EMS) survey scan were selected and fragmented in the collision cell and the fragments recorded following and enhanced product ion (EPI) scan.

### 5.12. Generation of C-Terminally Truncated Variants of MoxYAltN

Homology models of MoxYAltN and MoxYAltNC were generated using Yasara (version 18.4.24.L.64, Yasara Biosciences GmbH, Vienna, Austria). Ligand binding analysis was performed with LigPlot+ version 2.1 [60]. Structural alignments of the homology models of MoxYAltN and MoxYAltNC, as well as MoxYAltN with BVMOs of which the structures have been solved, were performed with UCSF Chimera software (1.11.2, University of San Francisco, CA, USA). The BVMOs used for comparison were PAMO from *Thermobifida fusca* (PDB ID 1W4X) [26], CHMO from *Rhodococcus* sp. HI-31 (PDB ID 3GWF) [27] and BVMO_AFL838_ from *Aspergillus flavus* (PDB ID 5J7X) [28]. C-terminally truncated mutants of MoxYAltN were generated at positions 501 (MoxYAltN_Tr501), 545 (MoxYAltN_Tr545) or 546 (MoxYAltN_Tr546), as well as a mutant from which a predicted loop spanning residues 528–541 was excised (MoxYAltN_Loop). All truncated mutants were generated using inverse PCR (primers shown in Table S4) as described for intron removal. Expression of the mutants were performed as described for the MoxY variants, and expression of the total and soluble fractions were evaluated with SDS-PAGE. Activity of the mutants were determined with whole-cell biotransformations using 10 mM phenylacetone, as described for the MoxY variants.

## Figures and Tables

**Table 1:** Whole-cell biotransformation of ketone substrates by MoxYAltN and MoxYAltNC. Conversion values are averages of duplicate experiments.

Substrate	Major Products	Conversion after 2 h (%)	
		MoxYAltN	MoxYAltNC
2-octanone	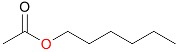	9.38 ± 0.17	0.82 ± 0.07
3-octanone	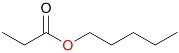	1.60 ± 0.04	0.33 ± 0.02
2-decanone	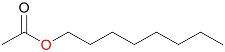	20.68 ± 0.12	2.24 ± 0.10
2-undecanone	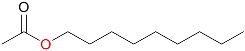	22.93 ± 2.35	3.20 ± 0.07
2-dodecanone	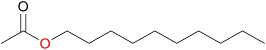	12.48 ± 2.52	3.71 ± 0.09
phenylacetone	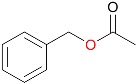	22.99 ± 0.60	2.57 ± 0.02
4-phenyl-2-butanone	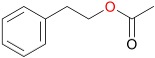	17.01 ± 0.40	1.99 ± 0.17
4-(4-hydroxyphenyl)-2-butanone	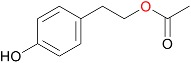	5.67 ± 0.19	0.80 ± 0.03
2-phenylcyclohexanone	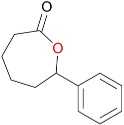	29.18 ± 1.52	4.31 ± 0.13
*rac*-bicyclo[3.2.0]hept-2-en-6-one	(Figure 7)	26.02 ± 0.31	4.01 ± 0.14

**Table 2:** Oligonucleotide primers used in this study.

Primer Pair No.	Primer Function	Sequence ^1^	Annealing Temperature
1	Amplification of the *moxY* gene from the gDNA of *A. flavus*	5′-CAT ATG GAC CCG GCC AAC CGC CCG TTG-3′	64 °C
		5′-CTC GAG CTA GCG GTT ACT GTC AGA AAC TCC ATT GG-3′	
2	Amplification of the *moxYAltNC* gene from the gDNA of *A. flavus*	5′-CAT ATG TCA AAG GTG GAC TAC TCT CAG CC-3′	60 °C
		5′-AAG CTT TTA CGT GAA ACG GAC AAG CGC-3′	
3	Removal of intron 1 from the pSMART:moxY and pSMART:moxYAltNC construct	5′-GTA CAA GAA CAA CGA GAC GGG TCG-3′	59 °C
		5′-CAG CTT CGG CAG TTA TCT TTC CAC AC-3′	
4	Removal of intron 2 from the pSMART:moxY and pSMART:moxYAltNC construct	5′-GTA CGA AGA GGT GGG CGG CAA TCC-3′	63 °C
		5′-CAT TTC GGG TCG ATC TCC TGT AAG CCC AG-3′	

^1^ Underlined sequences indicate introduced restriction sites for *Nde*I, *Hind*III, and *Xho*I.

## Supplementary Materials

The following are available online at http://www.mdpi.com/2072-6651/10/12/521/s1, Figure S1, SDS-PAGE analysis of the expression of the *moxY* coding sequence in *E. coli* BL21-Gold(DE3); Figure S2, Alignment of EST sequences of the *moxY* gene to the genomic DNA of *Aspergillus flavus* NRRL3357; Figure S3, SDS-PAGE analysis of the (A) total protein fraction and (B) soluble protein fraction of *E. coli* BL21-Gold(DE3) expressing the MoxY variants from the pET-28b(+) vector; Figure S4, A. Elution profile of MoxYAltN from the Superdex HR 200 column, showing two peaks, B. SDS-PAGE analysis of the two peaks; Figure S5, SDS-PAGE of the purification of MoxYAltNC.; Figure S6, Ketone substrate conversions evaluated by biotransformations with the MoxYAltN and NC; Figure S7, A. Superimposed structures of the homology model of MoxYAltN and MoxYAltNC B. Superimposed structures of the homology model of MoxYAltN and PAMO. C & D. Ligplot+ analysis for the homology models of MoxYAltN and MoxYAltNC; Figure S8, SDS-PAGE analysis of the (A) total protein fraction and (B) soluble protein fraction of *E. coli* BL21-Gold(DE3) co-expressing the truncated mutants of MoxYAltN from the pET-28b(+) vector with the GroES/EL chaperone from the pGro7 vector; and the (C) total protein fraction and (D) soluble protein fraction of *E. coli* BL21-Gold(DE3) expressing the truncated mutants of MoxYAltN from the pET-28b(+) vector; Table S1. Whole-cell conversions of ketone substrates by MoxYAltN and MoxYAltNC after 2 h; Table S2. GC programs for the analysis of biotransformations of ketone substrates; Table S3. GC program for the separation of *rac*-bicyclo[3.2.0]hept-2-en-6-one and products extracted from whole-cell biotransformations. Table S4. Primer sequences to create C-terminally truncated variants of MoxYAltN at residues 501, 545 and 546, and excision of a loop located at positions 526–541.
