# Supplementary material for: Alternative Splicing of the Aflatoxin-Associated Baeyer–Villiger Monooxygenase from Aspergillus flavus: Characterisation of MoxY Isoforms
Source: Toxins (Basel). 2018 Dec 5;10(12):521. doi: 10.3390/toxins10120521 (PMC6315744; doi:10.3390/toxins10120521)
Supplement: Supplementary file 1 [file toxins-10-00521-s001.pdf]

Supplementary Materials: Alternative Splicing of the Aflatoxin-Associated Baeyer-Villiger Monooxygenase from *Aspergillus flavus*: Characterisation of MoxY Isoforms

Carmien Tolmie, Martha S. Smit and Diederik J. Opperman

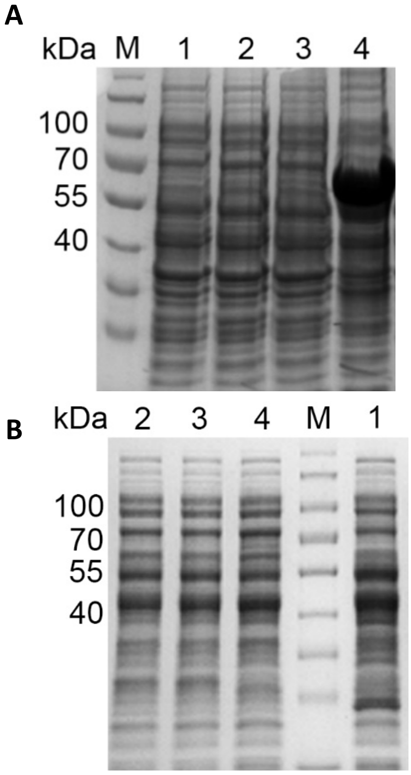

Figure S1. SDS-PAGE analysis of the expression of the *moxY* coding sequence in *E. coli* BL21-Gold(DE3). (**A**) Total protein fraction; (**B**) soluble fraction obtained by lysozyme lysis and centrifugation at 7000× *g* for 30 min. M, PageRuler™ Prestained protein ladder; lane 1, pET-22b(+) empty vector control; lane 2, pET-22b(+):moxY; lane 3, pET-22b(+):moxY-CTH; lane 4, pET-28b(+):moxY.

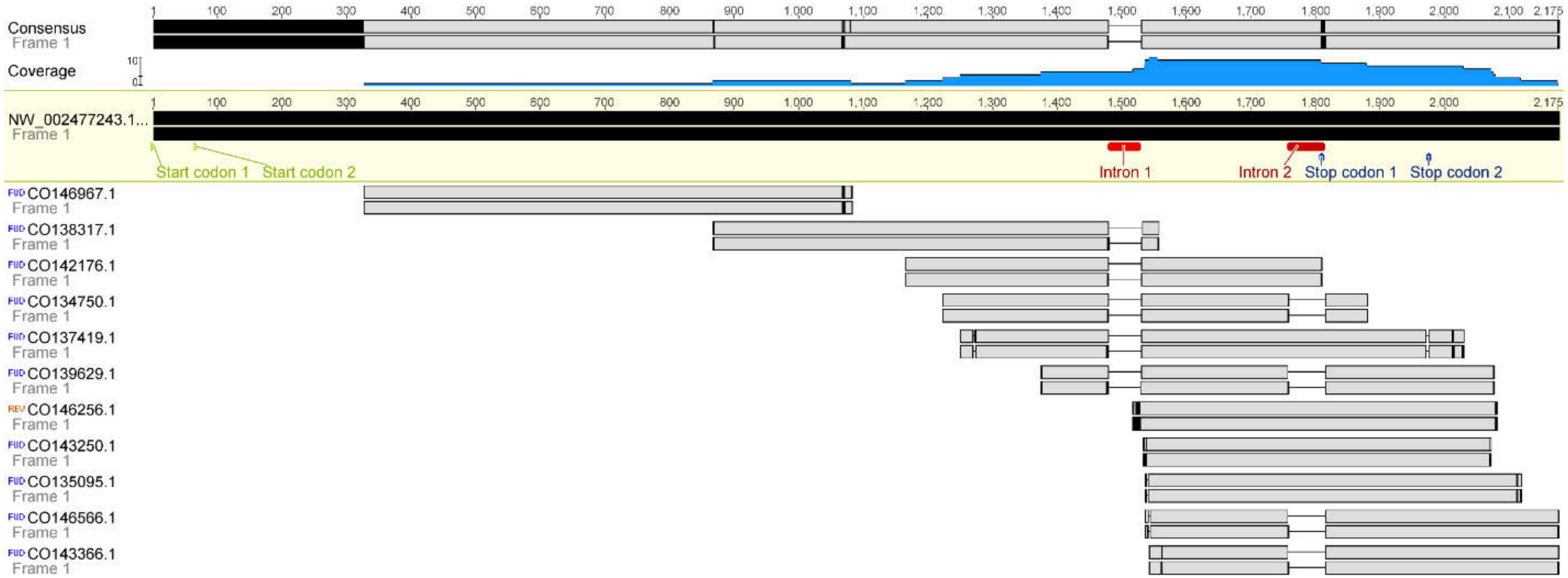

Figure S2. Alignment of the EST sequences of the *moxY* gene to the genomic DNA of *Aspergillus flavus* NRRL3357. A second intron is spliced out in about half of the EST sequences, removing the first stop codon and creating an alternative and elongated C-terminus. No EST sequence is available that covers the N-terminus to indicate the location of the true start codon. The alignment and image were generated using Geneious software version 7.1.3.

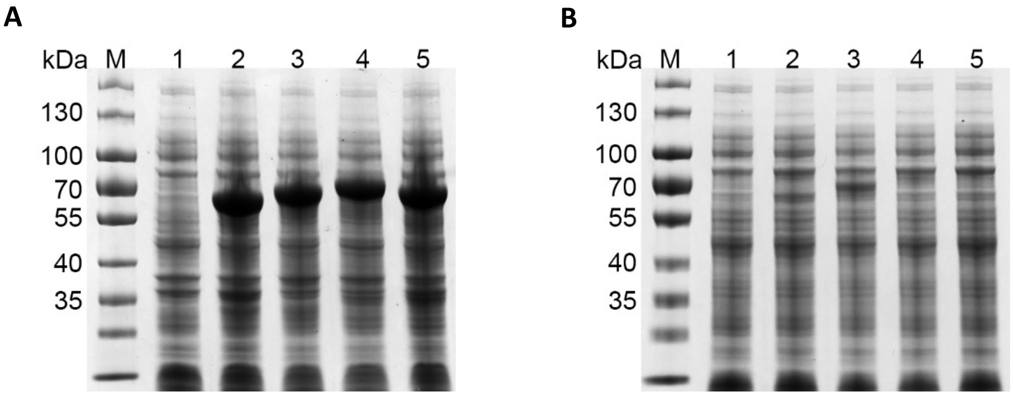

Figure S3. SDS-PAGE analysis of the (**A**) total protein fraction and (**B**) soluble protein fraction of *E. coli* BL21-Gold(DE3) expressing the MoxY variants from the pET-28b(+) vector. M, PageRuler Prestained protein ladder; 1, pET-28b(+) empty vector control; 2, MoxY (66.5 kDa); 3, MoxYAltN (69.0 kDa); 4, MoxYAltNC (73.1 kDa); 5, MoxYAltC (70.7 kDa).

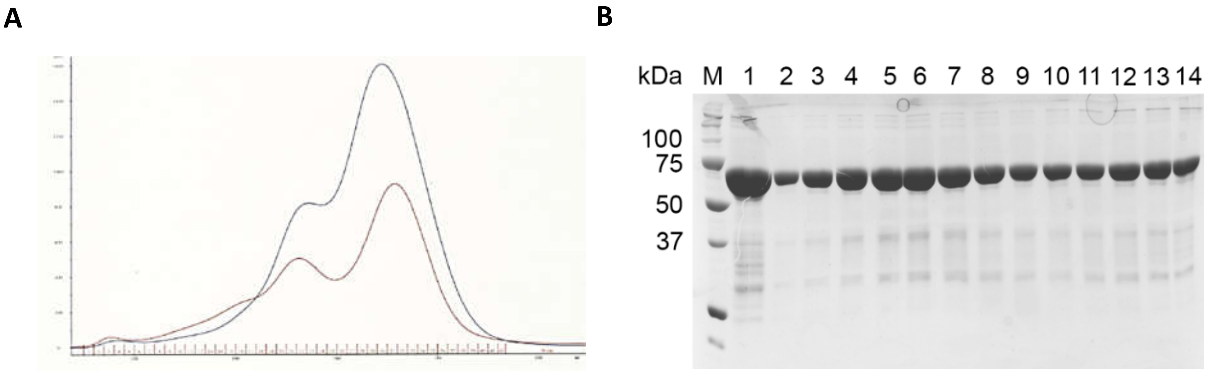

Figure S4. (**A**) Elution profile of MoxYAltN from the Superdex HR200 column, showing two peaks. (**B**) SDS-PAGE analysis of the two peaks. M, molecular weight marker; lane 1, ultracentrifuged fraction, lanes 2–14, gel-filtration fractions of the two peaks showing identical profiles.

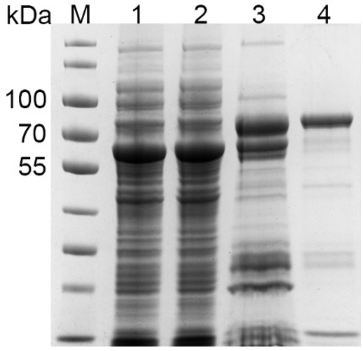

Figure S5. SDS-PAGE of the purification of MoxYAltNC. M, PageRuler Prestained protein ladder; lane 1, soluble fraction; lane 2, ultracentrifuged fraction; lane 3, pooled fractions after IMAC; lane 4, pooled fractions after anion-exchange chromatography.

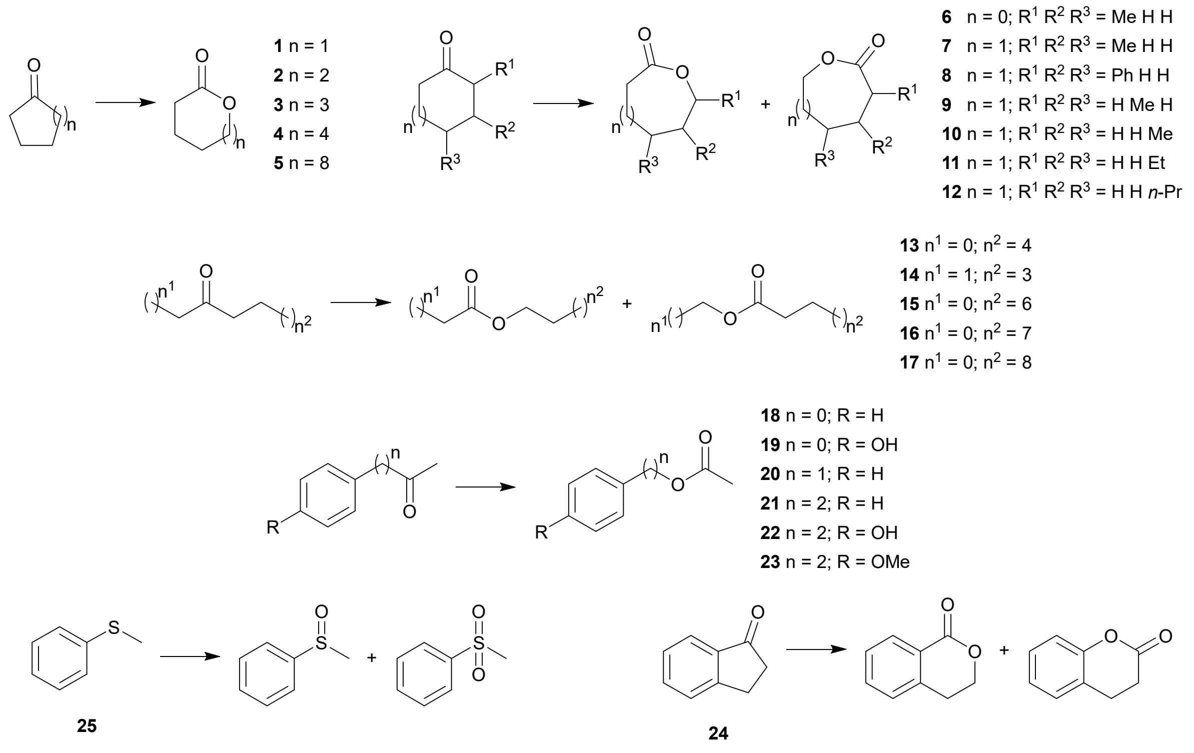

Figure S6. Ketone substrate conversions evaluated by biotransformations with MoxYAltN and MoxYAltNC.

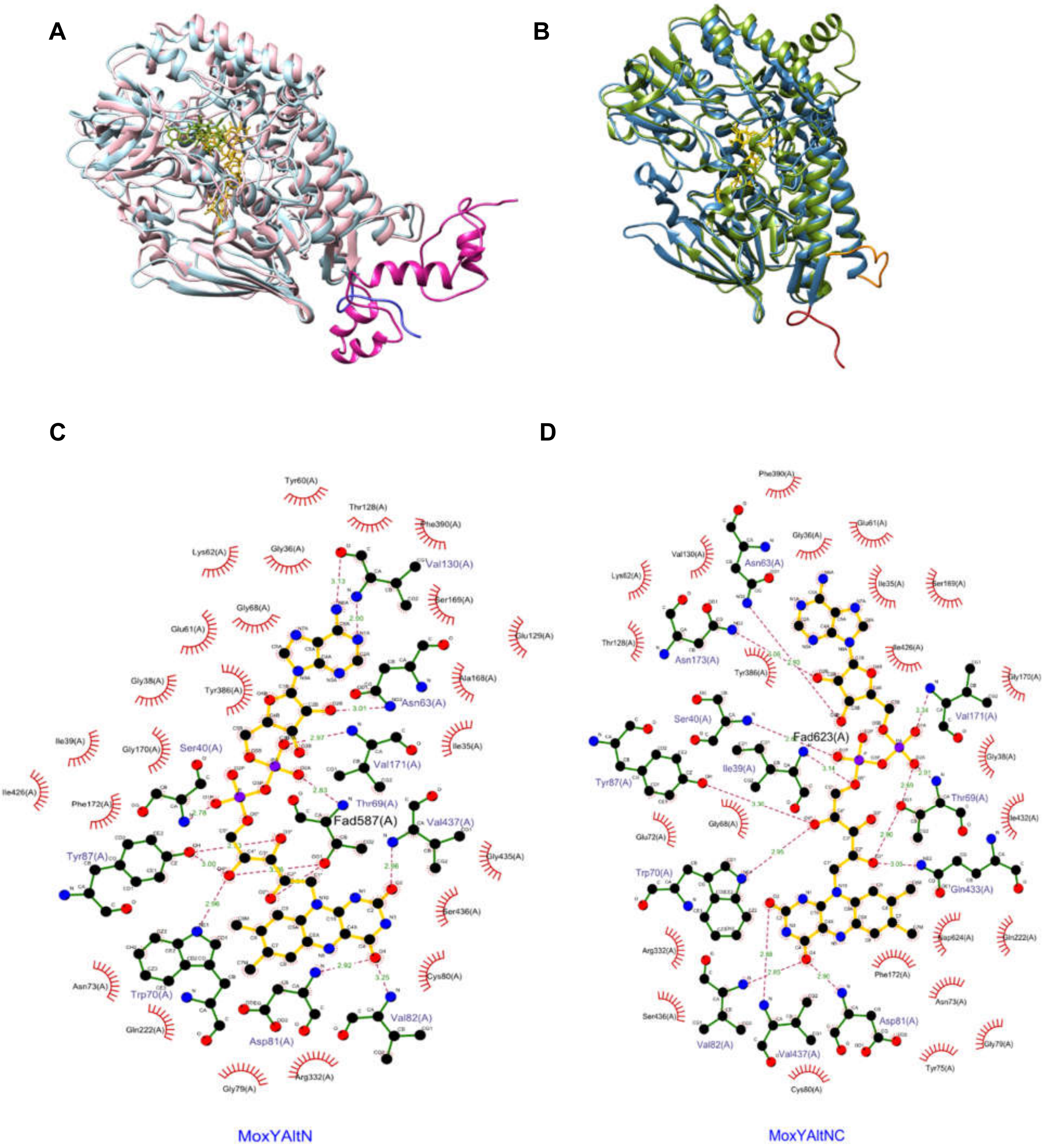

Figure S7. (**A**) Superimposed structures of the homology model of MoxYAltN, shown in light blue, and MoxYAltNC, shown in pink. The C-terminus of MoxYAltN is shown in medium blue and the C-terminus of MoxYAltNC is shown in magenta. (**B**) Superimposed structures of the homology model of MoxYAltN, shown in blue, and PAMO, shown in green. The unstructured loop near the C terminus of MoxYAltN is shown in orange, while the elongated C-terminus is shown in red. (**C**) & (**D**) Ligplot+ analysis for the homology models of MoxYAltN and MoxYAltNC.

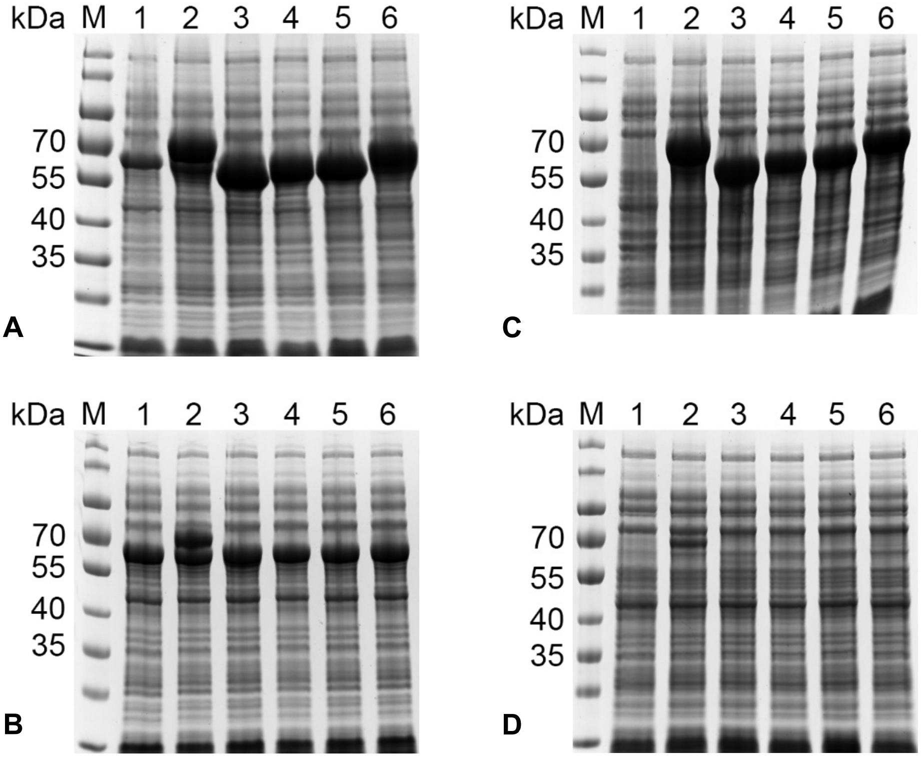

Figure S8. SDS-PAGE analysis of the (**A**) total protein fraction and (**B**) soluble protein fraction of *E. coli* BL21-Gold(DE3) co-expressing the truncated mutants of MoxYAltN from the pET-28b(+) vector with the GroES/EL chaperone from the pGro7 vector; and the (**C**) total protein fraction and (**D**) soluble protein fraction of *E. coli* BL21-Gold(DE3) expressing the truncated mutants of MoxYAltN from the pET-28b(+) vector. M, PageRuler Prestained protein ladder; 1, pET-28b(+) empty vector control; 2, MoxYAltN wild-type (66.5 kDa); 3, MoxYAltN_Tr501 (59.2 kDa); 4, MoxYAltN_Tr545 (64.4 kDa); 5, MoxYAltN_Tr546 (64.5 kDa); 6, MoxYAltN_Loop (69.0 kDa). The GroEL component of the GroES/EL chaperone can be seen as a band of approximately 60 kDa in gels A and B.

Table S1. Whole-cell conversions of ketone substrates by MoxYAltN and MoxYAltNC after 2 hours.

Substrate No.	Substrate	MoxYAltN	MoxYAltNC
1	Cyclopentanone	-	-
2	Cyclohexanone	-	-
3	Cycloheptanone	-	-
4	Cyclooctanone	-	-
5	Cyclododecanone	-	-
6	2-methylcyclopentanone	-	-
7	2-methylcyclohexanone	-	-
8	2-phenylcyclohexanone	+++	+
9	3-methylcyclohexanone	-	-
10	4-methylcyclohexanone	-	-
11	4-ethylcyclohexanone	-	-
12	*n*-propylcyclohexanone	-	-
13	2-octanone	++	+
14	3-octanone	+	+
15	2-decanone	+++	+
16	2-undecanone	+++	+
17	2-dodecanone	++	+
18	Acetophenone	-	-
19	4-hydroxyacetophenone	-	-
20	Phenylacetone	+++	+
21	4-phenyl-2-butanone	+++	+
22	4-(4-hydroxyphenyl)-2-butanone	++	+
23	4-(4-methoxyphenyl)-2-butanone	-	-
24	1-indanone	-	-
25	thioanisole	-	-

+++: ≥15% conversion, ++: =5–15% conversion, +: ≤5% conversion, -: =no conversion.

Table S2. GC programs for the analysis of biotransformations of ketone substrates using a Finnigan TRACE GC Ultra (Thermo Fisher Scientific) equipped with a FactorFour™ VF-5ms column (60 m × 0.25 mm × 0.25 µm, Agilent Technologies, coupled to a Mass Spectrometer (MS). Substrates shown in bold were converted by MoxYAltN and MoxYAltNC.

**Compound**	**Program [a]**	**Retention time (min) Substrates**	**Retention time (min) Products [b]**
Cyclopentanone	80/2/15/185/0	1.92	6.03
Cyclohexanone	60/1/10/110/4/25/200/2	2.82	6.2
Cycloheptanone	60/1/10/110/4/25/200/2	3.82	4.56
Cyclooctanone	60/1/10/110/4/25/200/2	5.05	5.2
Cyclododecanone	80/2/15/250/0	8.92	
2-methylcyclopentanone	80/2/15/250/0	2.01	4.67(D) 4.71 (P)
2- methylcyclohexanone	60/1/10/110/4/25/200/2	3.47	6.83 (D) 6.90 (P)
2-phenylcyclohexanone	80/2/8/140/0/15/220/2	14.25	15.57
3-methylcyclohexanone	60/1/10/110/4/25/200/2	3.53	7.20 (D) 7.35 (P)
4-methylcyclohexanone	60/1/10/110/4/25/200/2	3.6	7.46
4-ethylcyclohexanone	60/1/10/110/4/25/200/2	5.16	10.62
n-propylcyclohexanone	60/1/10/110/4/25/200/2	6.66	12.01
2-octanone	80/2/15/250	4.28	4.21 (P)
3-octanone	80/2/15/250	4.23	4.35 (D) 4.42 (P)
2-decanone	80/2/15/250	6.51	6.65 (P) 6.73 (D)
2-undecanone	80/2/15/250	7.54	7.66 (P) 7.74 (D)
2-dodecanone	80/2/15/250	8.49	8.58 (P) 8.72 (D)
Acetophenone	60/2/8/140/0/15/220/2	11.65	10.62
Phenylacetone	80/2/8/140/0/15/220/2	7.17	7.75
4-phenyl-2-butanone	80/2/8/140/0/15/220/2	9.25	9.39
4-(4-hydroxyphenyl-2-butanone)	60/15/5/160/25/250/2	24.56	24.67
4-(4-methoxyphenyl-2-butanone)	80/2/8/140/0/15/220/2	12.91	12.99
1-indanone	60/2/5/165/0	12.85	15.57
rac-bicyclo[3.2.0]hept-2-en-6-one	80/2/15/260/5	3.85	6.56 (D) 6.58 (P)
Thioanisole	60/2/8/140/0/15/220/2	6.52	10.83, 11.85 [c]

[a] Initial temp (°C)/ time (min)/ slope (°C/min)/ temperature (°C)/ time (min)/ slope (°C/min)/ temperature (°C)/ time), [b] P = proximal product, D = distal product, [c] Sulfoxide, sulfone.

Table S3. GC program for the separation of *rac*-bicyclo[3.2.0]hept-2-en-6-one and products extracted from whole-cell biotransformations. A Finnigan TRACE GC Ultra (Thermo Scientific) equipped with an Astec CHIRALDEX™ G-TA column (30 m × 0.25 mm × 0.12 µm, Sigma Aldrich) was used and compounds were detected by FID. Retention times for the substrates and products are indicated.

Program [a]	Retention time (min)	Compound	
80/7/10/125/15/15/160/1	6.7	(-)-(1*S*,5*R*)-bicyclo[3.2.0]hept-2-en-6-one	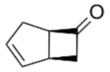
	7.3	(+)-(1*R*,5*S*)-bicyclo[3.2.0]hept-2-en-6-one	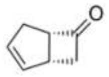
	21.7	(-)-(1*R*,5*S*)-3-oxabicyclo[3.3.0]oct-6-en-2-one	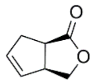
	22.5	(+)-(1*R*,5*S*)-2-oxabicyclo[3.3.0]oct-6-en-3-one	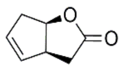
	24.4	(-)-(1*S*,5*R*)-2-oxabicyclo[3.3.0]oct-6-en-3-one	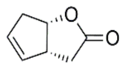
	25.0	(+)-(1*S*,5*R*)-3-oxabicyclo[3.3.0]oct-6-en-2-one	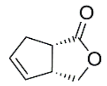

[a] Initial temp (°C)/ time (min)/ slope (°C/min)/ temperature (°C)/ time (min)/ slope (°C/min)/ temperature (°C)/ time.

Table S4. Primer sequences to create C-terminally truncated variants of MoxYAltN at residues 501, 545 and 546, and excision of a loop located at positions 526–541.

Primer	Sequence	Annealing Temperature (°C)
Truncation of C-terminus:		
MoxY_TrpET28_F	TAG CTC GAG CAC CAC CAC CAC CAC C	66.4
MoxY_Tr545_R	CTG GAT TGT CCA GCC CAT GCC CAG G	65.8
MoxY_Tr501_R	CCG ACC CGT CTC GTT GTT CTT GTA CC	62.7
MoxY_Tr546_R	GTC CTG GAT TGT CCA GCC CAT GCC C	65.7
Excision of C-terminal loop:		
MoxY_Loop_G541_F	GGC TGG ACA ATC CAG GAC CGC AAA G	64.0
MoxY_Loop_A526_R	GTC GAA GTC TTC GTA GCG AGG CTG GTC	63.8
